# Unique mitochondrial DNA lineages in Irish stickleback populations: cryptic refugium or rapid recolonization?

**DOI:** 10.1002/ece3.853

**Published:** 2014-05-21

**Authors:** Mark Ravinet, Chris Harrod, Christophe Eizaguirre, Paulo A Prodöhl

**Affiliations:** 1 Institute for Global Food Security, School of Biological Sciences, Queen's University BelfastBelfast, U.K; 2 Lovén Centre-Tjärnö, Department of Biology and Environmental Sciences, University of GothenburgGothenburg, Sweden; 3 Instituto de Ciencias Naturales Alexander Von Humboldt, Universidad de AntofagastaChile; 4 GEOMAR|Helmholtz Centre for Ocean ResearchDuesternbrooker weg 20, 24105, Kiel, Germany; 5 Max Planck Institute for Evolutionary BiologyAugust Thienemannstr. 2, 24306, Ploen, Germany

**Keywords:** ABC, anadromous fish, British Isles, phylogeographical hypothesis testing, statistical phylogeography

## Abstract

Repeated recolonization of freshwater environments following Pleistocene glaciations has played a major role in the evolution and adaptation of anadromous taxa. Located at the western fringe of Europe, Ireland and Britain were likely recolonized rapidly by anadromous fishes from the North Atlantic following the last glacial maximum (LGM). While the presence of unique mitochondrial haplotypes in Ireland suggests that a cryptic northern refugium may have played a role in recolonization, no explicit test of this hypothesis has been conducted. The three-spined stickleback is native and ubiquitous to aquatic ecosystems throughout Ireland, making it an excellent model species with which to examine the biogeographical history of anadromous fishes in the region. We used mitochondrial and microsatellite markers to examine the presence of divergent evolutionary lineages and to assess broad-scale patterns of geographical clustering among postglacially isolated populations. Our results confirm that Ireland is a region of secondary contact for divergent mitochondrial lineages and that endemic haplotypes occur in populations in Central and Southern Ireland. To test whether a putative Irish lineage arose from a cryptic Irish refugium, we used approximate Bayesian computation (ABC). However, we found no support for this hypothesis. Instead, the Irish lineage likely diverged from the European lineage as a result of postglacial isolation of freshwater populations by rising sea levels. These findings emphasize the need to rigorously test biogeographical hypothesis and contribute further evidence that postglacial processes may have shaped genetic diversity in temperate fauna.

## Introduction

Repeated recolonization of freshwater environments after Pleistocene glaciations has played a major role in shaping the evolution and distribution of numerous anadromous taxa (Hewitt 1996, 2001; Avise 2000). Located at the western fringe of Northern Europe, Ireland is likely to have been one of the first regions recolonized by anadromous species from the North Atlantic, following ice retreat after the last glacial maximum (LGM). The region is additionally a zone of secondary contact for divergent evolutionary lineages in these species, that is, genetically divergent monophyletic units arising from allopatry (Consuegra et al. 2002; Jordan et al. 2005; McKeown et al. 2010). The presence of divergent mitochondrial haplotypes, absent from populations in both Britain and Northern Europe, suggests that postglacial recolonization of some species may have occurred from a cryptic refugium (e.g., *Salmo trutta* L., McKeown et al. 2010; *Gammarus duebeni,* Krebes et al. 2010). Research on anadromous fish phylogeography in Ireland thus provides an opportunity to test whether such a cryptic northern refugium played a role in recolonization and local species evolution.

Ireland's glacial history has played a major role in shaping its biogeography. Geographical isolation by rapidly rising sea levels during the late Pleistocene prevented recolonization by obligate freshwater fishes, producing a depauperate, largely euryhaline native fish fauna (Wheeler 1977; Griffiths 1997). Similarly, the occurrence of endemic “glacial relict” taxa such as the Irish pollan – *Coregonus pollan –* suggests a novel recolonization history within Europe (Harrod et al. 2002; Ferguson 2004; Audzijonyte and Väinola 2006). The presence of unique evolutionary lineages – that is, monophyletic lineages found only in Irish populations – in anadromous species in Ireland furthermore raises the possibility that some species may have recolonized from a cryptic refugium close the present-day Irish landmass (Stewart and Lister 2001; Maggs et al. 2008; McKeown et al. 2010).

**Figure 1:**
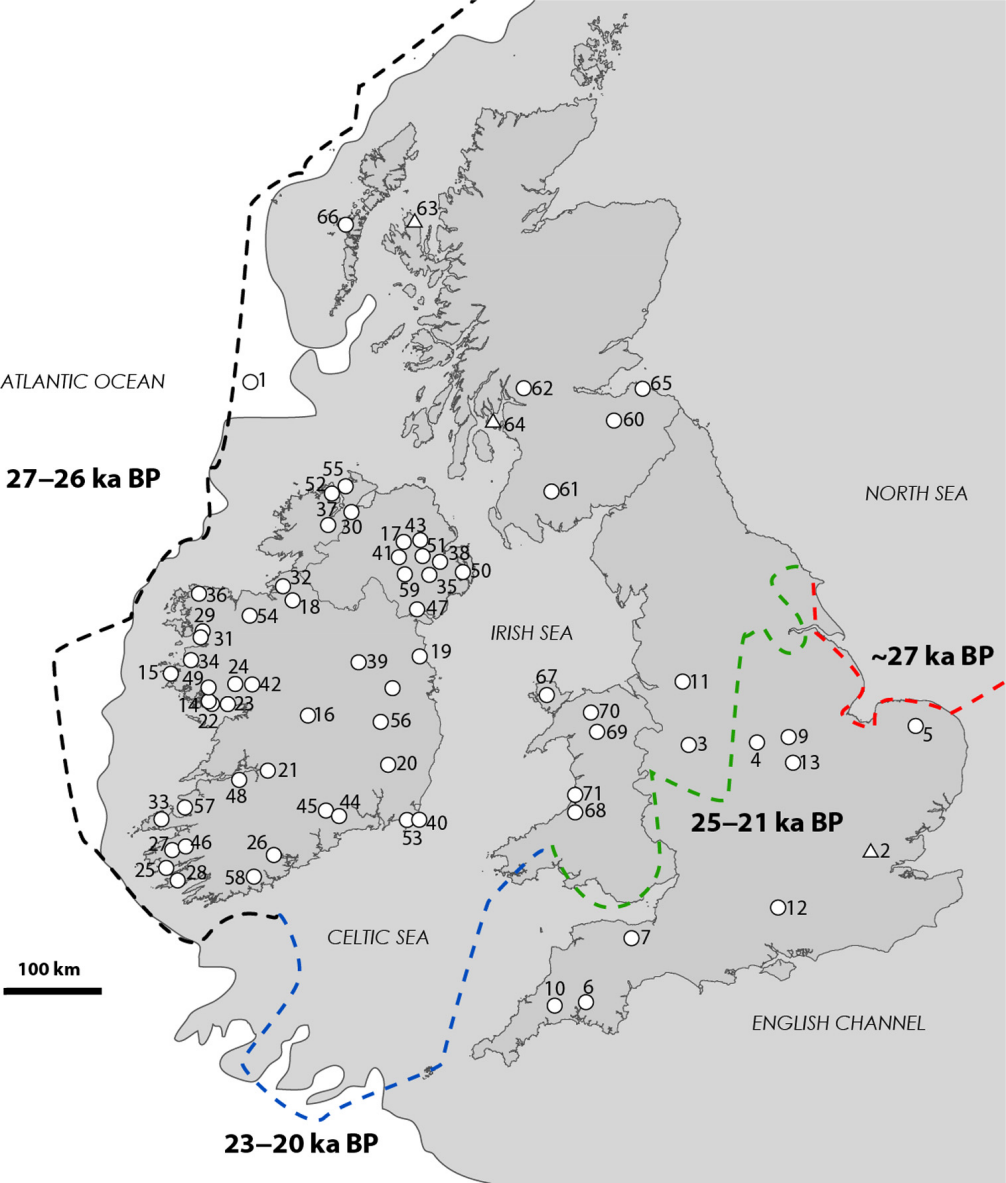
Location of sample sites throughout Britain and Ireland. Circles represent sites sampled by authors, and triangles represent sites sampled by Mäkinen and Merilä (2008). Dark shading represents putative paleocoastline at LGM (adapted from Clark et al. 2012). Colored dashed lines represent approximate maximum extent of British–Irish Ice Sheet margins with estimates of timings taken from youngest advance date and oldest deglaciation date (also adapted from Clark et al. 2012). Numbers refer to sampling sites listed in Table S1.

Testing whether such a cryptic refugium played a role in the recolonization of Ireland has been challenging due to a previous lack of consensus on ice sheet extent (Greenwood and Clark 2009; Chiverell et al. 2010). Although it is well established that Ireland and Britain were dominated by the British–Irish Ice Sheet (BIIS), early studies suggested that the southwest and western parts of Ireland were ice-free at the LGM (Charlesworth 1928a,b1928b; Synge 1981; Bowen et al. 2002). Recent research, however, provides reliable evidence for greater ice extent and more robust timings (see Fig. 1), turning the BIIS into one of the best understood and well-resolved ice sheets in Europe (Clark et al. 2012). Such high-resolution glacial history now offers an opportunity to explicitly test whether a cryptic refugium existed and if so, what role it played in recolonization (Knowles and Maddison 2002; Knowles 2009). Yet to date, few phylogeographical studies have been carried out.

The three-spined stickleback (*Gasterosteus aculeatus*) is one of the few native fish species in Ireland and is present in both marine and freshwater habitats throughout the region (Ravinet et al. 2013). Throughout its distribution, the evolutionary diversification of the stickleback species complex is closely linked to the climatic oscillations of the Pleistocene, with freshwater populations being established by marine ancestors following deglaciation (Bell and Foster 1994; McKinnon and Rundle 2002). Within Europe, high levels of genetic diversity and divergence exist between southern populations in the Balkan Peninsula and the Black Sea, suggest a more ancient origin of stickleback populations in these regions (Mäkinen and Merilä 2008; DeFaveri et al. 2012a). In contrast, Northern European populations in formerly glaciated regions exhibit lower genetic diversity but still harbor multiple divergent mitochondrial lineages as a result of recolonization from multiple marine sources following the LGM (Mäkinen and Merilä 2008). Given this, the three-spined stickleback provides an excellent biological model to test for the existence of a cryptic refugium and its likely role in the recolonization of Ireland by anadromous species.

To date, very little is known of the genetic structuring and diversity present in Irish stickleback populations. Our first objective was therefore to investigate the evolutionary history of stickleback populations in Ireland. In short, we used mitochondrial DNA to establish whether Ireland harbored unique haplotypes and divergent lineages occurred in secondary contact in the region. We also investigated whether microsatellite data reflected large-scale mitochondrial divergence or regional clustering based on contemporary gene flow and drift as a result of postglacial isolation. An additional objective was to test models of stickleback recolonization in Ireland from multiple marine sources, using timings from the revised BIIS model within an approximate Bayesian computation (ABC) framework. Specifically, we tested whether divergence and the presence of a third evolutionary lineage unique to Ireland was consistent with the hypothesis of a spread from a cryptic Irish refugium based on divergence times and effective population size.

## Materials and Methods

### Sample collection

In order to obtain a reliable characterization of genetic diversity of three-spined stickleback populations in Ireland, we carried out an extensive sampling programme. The majority of sampling was therefore conducted at 46 sites located across the island of Ireland (Fig. 1, Table S1). Given the close proximity of Ireland to Britain and their shared glacial history, we also collected additional samples from sites located in England, Scotland, and Wales (12, 7, and 5 sites, respectively, see Fig. 1, Table S1). Sampling took place at all 68 sites between April 2008 and October 2010 (Fig. 1, Table S1). Sticklebacks (*n* = 730) were captured using minnow traps, beach seines, dip nets, and electrofishing, euthanized using an overdose of MS-222 or clove oil, and then placed immediately in 100% ethanol. An additional sample of marine sticklebacks from the North Atlantic Ocean was obtained during a research trawl by the Irish Marine Institute research vessel the R.V. Celtic Voyager in order represent a purely marine population (Site 1, Fig. 1).

### DNA extraction and PCR amplification

Deoxyribonucleic acid was extracted from caudal fin clips using Qiagen DNeasy tissue kits or following a salt extraction method (Aljanabi and Martinez 1997). DNA from a subset of individuals (*n* = 480), representing the 68 sample sites, was amplified for two mitochondrial regions, cytochrome B (forward 5′ ATGAAACTTTGGTTCCCCTCC 3′, reverse 5′ CGCTGAGCTACTTTTGCATGT 3′), and a partial control region sequence (forward 5′ CCTTTAGTCCTATAATGCATG 3′, reverse 5′ CCGTAGCCCATTAGAAAGAA 3′, taken from Mäkinen and Merilä (2008). We did not use the nested primer method they applied. Initial testing involving PCR replicates indicated that resulting by-directional sequencing data were of very good quality (i.e., no base call ambiguities); hence, we found no need to use nested approach for the current dataset. For both primer sets, PCR was carried out in 50 μL volume as follows: 2.5 mM MgCl_2_, 1 x PCR Buffer (Invitrogen, Carlsbad, CA), 200 μM dNTPs, 1 μL of each primer (10 picomoles), 2U *Taq* polymerase (Invitrogen), and approximately 20 ng stock DNA. For cyt B, thermocycling conditions consisted of one denaturation step at 94°C for 3 min, followed by 35 cycles of 30 s at 94°C, 30 s at 62°C, 60 s at 72°C, and then a final extension step at 72°C for 45 min. Similar conditions were used for control region, although the annealing temperature was set to 52°C. Products were purified and then sequenced using the Big Dye v3.1 cycle sequencing kit (Applied Biosystems Inc., Foster City, CA) prior to analysis on a 96 capillary ABI 3730XL DNA Analyzer (Applied Biosystems Inc). Sequence base calls were obtained using SEQUENCE ANALYZER v5.4 (Applied Biosystems Inc.) and were manually checked and edited using ChromasPro v1.42 and aligned with BioEdit v7.0.5.3.

Seven hundred and thirty samples from across all 69 sampling locations were amplified for nine microsatellite loci in two multiplex PCR cocktails after Kalbe et al. (2009, see S3 for further details). PCR amplification was carried out using Top-Bio PPP mastermix (Top-Bio, Czech Republic); total reaction volume was 3.5 μL with 1.5 μL mastermix, 1 μL template DNA (1–5 ng), and 0.035 μL (10 pM) of each primer with the remainder volume made up with ddH_2_0. Identical thermocycler conditions were used for both multiplexes as follows: 110°C heated lid, denaturation at 95°C for 15 min and then 20 cycles of 95°C for 30 s, 57°C for 1.5 min, and 72°C for 1.5 min, with a final extension of 60°C for 30 min. Fragment analysis was then performed on a 96 capillary ABI3730XL DNA Analyzer (Applied Biosystems Inc.). Raw fragment profiles for each individual were then manually genotyped using GENEMAPPER v4.1 (Applied Biosystems Inc.).

### Mitochondrial DNA analysis

Sequences for cytochrome B and control region were aligned and then concatenated (858 and 171 bp respectively, total length 1029 bp). Additional sequences from three UK populations (QUI, MEA, ALE – see Table S3) from Mäkinen and Merilä (2008) were also included alongside three outgroup haplotypes from Japanese Pacific populations (J. DeFaveri, University of Helsinki, personal communication) for comparative analysis. Molecular diversity indices including haplotype diversity (hd), nucleotide diversity (π), and two mutation-drift equilibrium statistics, Tajima's D and Fu & Li's F, were estimated first for population samples grouped by geographical region (i.e., England, Ireland, Scotland, Wales; see Table S2) and then by mitochondrial lineage (inferred from phylogeny and network analysis) using DnaSP v5 (Librado and Rozas 2009).

### Mitochondrial phylogeny and haplotype network

Our first aim was to use mitochondrial DNA to examine relationships between populations in Ireland and Britain and to examine how these grouped with previously described European mitochondrial lineages. Therefore, we constructed two mitochondrial datasets: The first included all unique haplotypes found in Ireland and Britain, and the second included these haplotypes alongside all of those from the European and Trans-Atlantic lineages described by Mäkinen and Merilä (2008; GenBank accession numbers EF525391-EF525449, see S3a and S3b). For outgroups, we used corresponding sequences from Pacific *G. aculeatus* and three sequences from *Gasterosteus wheatlandi* (GenBank accession numbers: AB445129, AF356078 and NC_011570).

**Figure 2:**
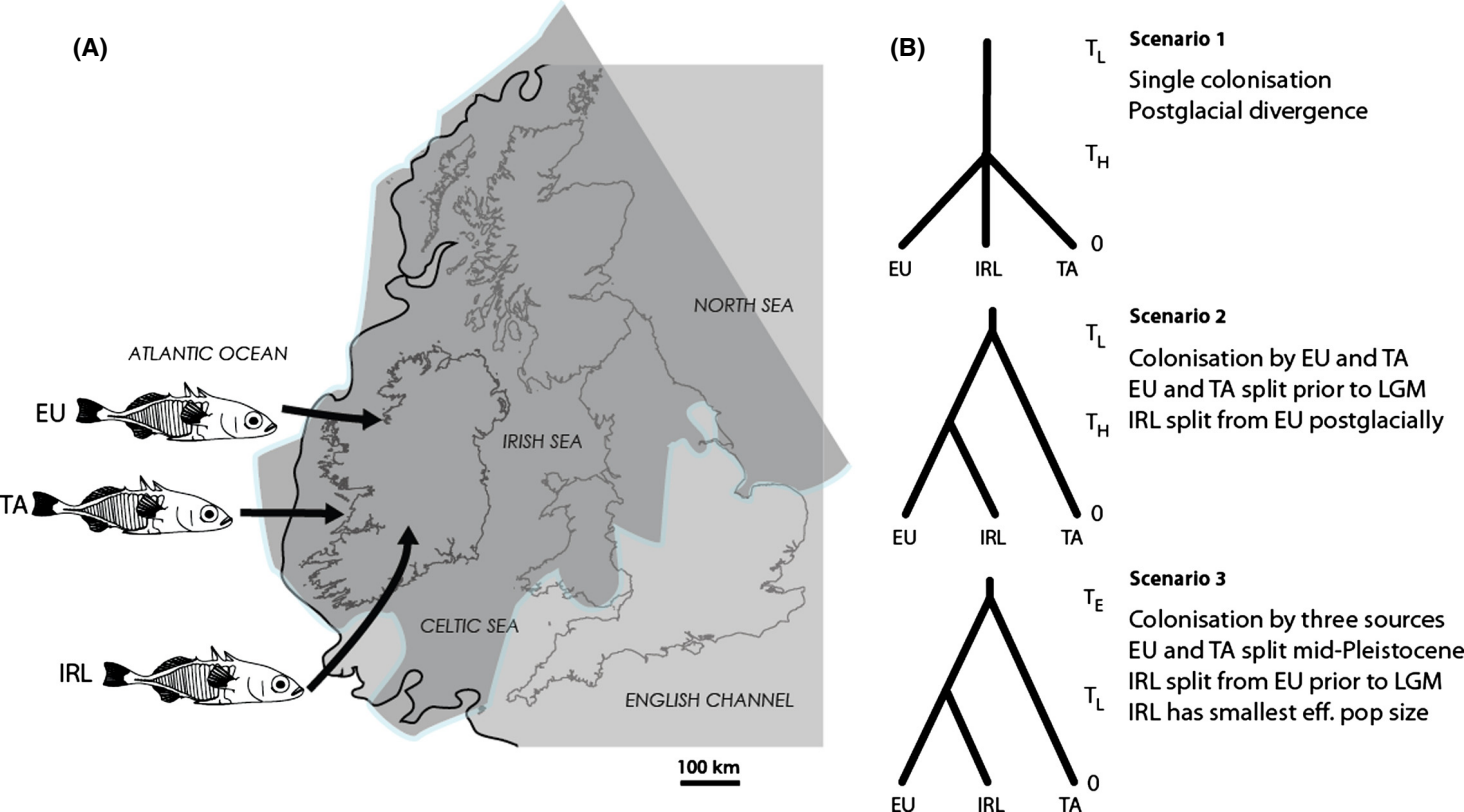
Hypothesized scenarios for postglacial recolonization of Ireland; (A) Map of British Isles indicating putative sources of recolonization; dark grey shaded area represents British–Irish Ice Sheet, and black line represents putative palaeocoastline at the LGM (both adapted from Clark et al. 2012); (B) Hypothesized scenarios used in ABC analysis, *T*_E_, *T*_L_, and *T*_H_ refer to divergence times during the Eemian, LGM and Holocene, respectively.

To examine the evolutionary relationships between haplotypes, we used a Bayesian phylogenetic approach implemented in mrBayes 3.1.2 (Ronquist and Huelsenbeck 2003). The most suitable model of sequence evolution, TN93 + Ι + Γ, was first established for both datasets using jModelTest, with model selection via Akaike information criteria (Posada 2008). For phylogeny, identical conditions were used for each dataset and Bayesian searches were initiated using the HKY + Ι + Γ model using *G. wheatlandi* as an outgroup; this model was used as the TN93 + Ι + Γ is not currently implemented in mrBayes 3.1.2, and it was also supported by a low AIC score. A total of six independent runs were conducted using four parallel MCMC chains of 15 × 10^6^ generations per run with a 50% burn-in and a temperature of 0.04 for the heating scheme. To further assess convergence, parameter estimates and log likelihood values of each run were compared in Tracer v1.5 (Rambaut and Drummond 2009), and pairwise split frequencies between runs were examined using AWTY (Nylander et al. 2008). A 50% rule majority consensus tree was then drawn from the sampled trees.

Phylogenetic methods are not particularly suitable for examining contemporary populations with extant ancestral haplotypes and large sample sizes, as they do not incorporate frequency data and homoplasy may obscure relationships (Posada and Crandall 2001). To account for this, we also chose to construct a median-joining network for Irish and British haplotypes using NETWORK 4.6 (http://www.fluxus-engineering.com). To resolve ambiguities and reticulations in the network, the network was edited by hand following Pfenniger and Posada (2002).

### Divergence time estimates and historical demography

Population isolation has been one of the major genetic consequences of the Pleistocene glaciations, driving the evolution of separate lineages (Hewitt 2001; Nielsen and Wakeley 2001). To evaluate the roles of both isolation and migration in stickleback phylogeography, we used IMa2 to produce coalescent-based estimates of divergence time, effective population size, and migration from our mitochondrial dataset (Hey 2010). Analysis was carried out simultaneously on all three lineages with a geometric heating scheme comprising 20 chains and a burn-in of 500,000 steps. Three independent runs were conducted to ensure parameter estimates were consistent and each run was only stopped when the chains appeared fully mixed and a sufficient number of genealogies (∼20,000) had been sampled. Independent runs were then combined in a single likelihood analysis.

### Microsatellite analysis of population clustering

The high levels of polymorphism exhibited by microsatellite markers means that at low sample sizes, estimates of genetic distance or population structure may be biased (Takezaki and Nei 1996; Ruzzante 1998). However, our intention in this study was not to investigate clustering at the scale of individual sampling sites, but rather to examine broader, larger-scale population structuring occurring at the regional level within Ireland and Britain and to test whether this was concordant with divergent evolutionary lineages detected using mitochondrial DNA. Hence, an average of 11 individuals per sampling site (minimum = 4, maximum = 30; see Table S2) amplified for nine microsatellite loci was sufficient to achieve our goals.

To determine regional-scale population clustering, we used STRUCTURE 2.3.3 (Pritchard et al. 2000). To improve parameter estimate efficiency, the value of lambda was first estimated from the data (Pritchard and Wen 2004) before running ten iterations of each estimate of *k* (1–25) using the correlated allele frequencies model (Falush et al. 2003). For every run, a 100,000-step burn-in with a subsequent 100,000 MCMC steps was applied. The most probable value of *k* was then evaluated following Evanno et al. (2005).

AMOVA in Arlequin v3.5 (Excoffier et al. 1992) was used to determine how to best partition the observed genetic variance. An initial test for concordance with the mitochondrial dataset was performed with the expectation that a high proportion of genetic variance explained by lineage suggests considerable correspondence between the two datasets. The relative contributions of STRUCTURE-inferred clusters, habitat, region, marine drainage (see Table S1), and population in explaining genetic variance were also examined.

### Approximate Bayesian computation

Statistically evaluating multiple, realistic biogeographical scenarios has, until relatively recently, proved problematic. Most methods of testing evolutionary models make use of likelihood functions to estimate the probability of the data, conditional on a given parameter value (Bertorelle et al. 2010; Csilléry et al. 2010). While this is straightforward for simple models, the estimation of the likelihood function for complex scenarios is extremely difficult and often intractable (Csilléry et al. 2010). This has been a major issue for phylogeographical hypothesis testing, because the range and dimensionality of alternative hypotheses are often large and complex (Knowles 2009). The development of approximate Bayesian computation (ABC) has provided an alternative, powerful means of overcoming these issues (Bertorelle et al. 2010; Cornuet et al. 2010; Csilléry et al. 2010). The principles of ABC are straightforward: The method approximates the likelihood function by simulating data under a hypothesized scenario and then calculates a set of summary statistics. Summary statistics are then accepted or rejected based on a threshold distance from the same statistics estimated from the observed data; estimates of parameter values can then be obtained from accepted simulated datasets (Bertorelle et al. 2010; Csilléry et al. 2010). Furthermore, ABC can be used to evaluate alternative hypotheses by modelling datasets under multiple scenarios and calculating the proportion of accepted statistics for each scenario – that is, the posterior probability of a given scenario (Cornuet et al. 2008, 2010).

To test the hypothesis that stickleback populations in Ireland were in part established from a cryptic refugium in close proximity to the Irish landmass (Fig. 2), we used DIYABC v 1.0.4.46 with three observed datasets: combined mtDNA and microsatellites, mtDNA only, and microsatellites only (Cornuet et al. 2010). Determining the nature of a refugium for three-spined stickleback, a species with a diverse habitat range, is not a straightforward task. To date, the consensus of stickleback phylogeographical studies is that divergent mitochondrial haplotypes present in freshwater populations indicate colonization by different marine lineages that probably arose as a result of allopatric isolation in separate marine basins during Pleistocene glacial cycles (Mäkinen and Merilä 2008; Aldenhoven et al. 2010; DeFaveri et al. 2012a). With this in mind, our ABC approach does not test for the location or direction of recolonization from different refugia, but rather whether the current genetic patterns observed in Ireland are consistent with recolonization by separate stickleback lineages that diverged prior to the LGM (Fig. 2).

To evaluate different recolonization hypotheses, three putative scenarios were considered (Fig. 2). (1) Ireland was colonized from a single marine refugium and subsequent postglacial isolation has resulted in the three divergent lineages. Under this scenario, mitochondrial lineages do not represent separate refugial sources, instead divergence occurred *in situ* following the LGM. (2) Ireland was colonized from two marine sources (European and Transatlantic lineages) and a third (Irish) lineage has subsequently arisen due to postglacial isolation. This scenario is consistent with Mäkinen and Merilä (2008) in that two lineages found in Northern Europe diverged prior to the LGM and that both have recolonized Irish waterbodies. However, an Irish lineage has diverged postglacially from the European lineage. 3) Ireland was colonized by three sources including a putative Irish refugium that would have existed during the LGM. Previous phylogeographical studies on anadromous fish in Ireland have shown unique and highly divergent Irish haplotypes to be located in the south and western parts of the region (McKeown et al. 2010). The presence of an isolated marine basin, in close proximity to these regions, would therefore be reflected by the divergence of Irish lineages around the time of the LGM and furthermore by a small effective population size in relation to larger refugial sources, such as the North Atlantic Ocean.

**Figure 3:**
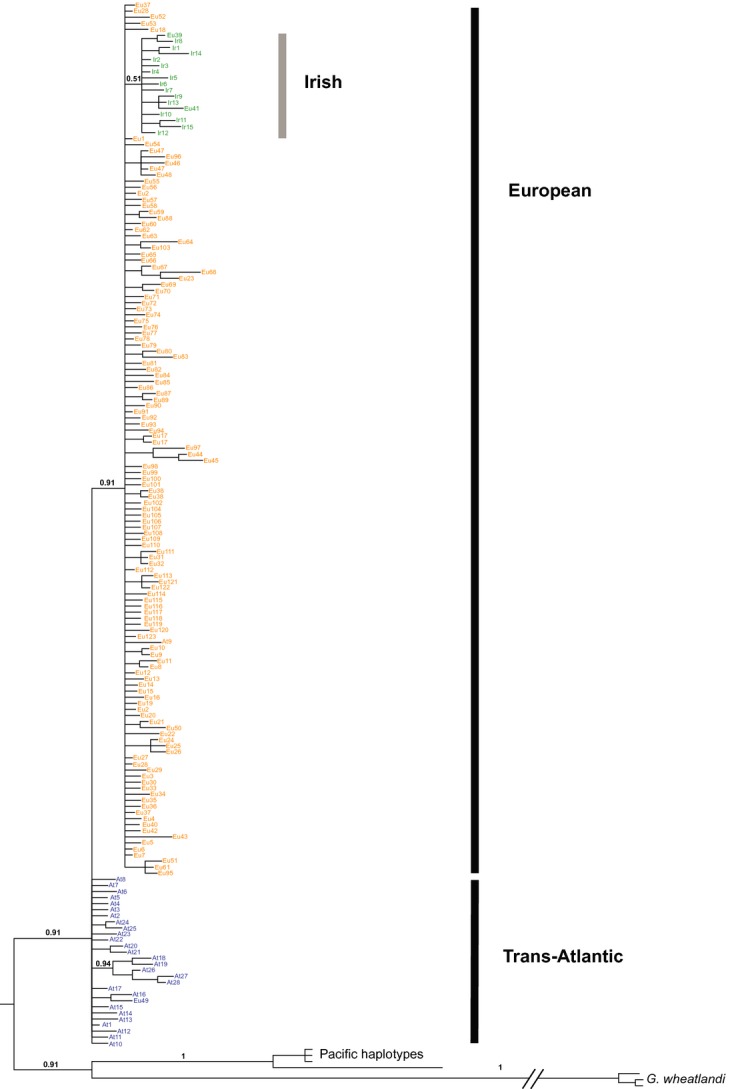
Fifty percent majority rule consensus phylogenies built using Bayesian inference for combined mitochondrial cytochrome B and control region haplotypes from Britain, Ireland, and Northern Europe. Haplotypes first described by Mäkinen and Merilä (2008) retain their original nomenclature. Branch labels indicate posterior probability support for major splits between lineages.

Priors for divergence times were based on the available literature for Ireland's glacial history (Clark et al. 2012) and using recently published dates for the LGM and Eemian interglacial from across Europe (Sejrup et al. 2005). Substitution rate and population size priors were set to encompass both our own estimates and those previously published (Mäkinen and Merilä 2008). For each scenario, a total of 1.06 × 10^6^ datasets were simulated and 30 summary statistics generated for each. Following this, posterior probabilities for each scenario were calculated using the logistic regression step (Cornuet et al. 2008). Confidence in model choice was assessed by simulating 1 500 (500 per scenario) pseudo-observed datasets, with parameter values drawn at random from the prior distributions of each scenario (see Table S5). To further quantify our confidence in model specification, rates of type I and type II error were calculated manually following the steps outlined by Cornuet et al. (2010).

## Results

### Mitochondrial DNA

A total of 364 individuals from Britain and Ireland were successfully amplified and sequenced for both mitochondrial regions. All resulting sequences were found to be of excellent quality, with very few ambiguous base calls. On the few instances where these were observed, the relevant samples were excluded from subsequent analyses. In total, 117 composite haplotypes (cyt B – 858 bp – and control region – 171 bp, total length – 1029 bp) were observed in the complete dataset. As we did not use the nested primer approach of Mäkinen and Merilä (2008), we performed an *in silico* test to check that the reduced sequence fragment did not lower our power to distinguish haplotypes. Trimming all sequences from the European and Trans-Atlantic lineages reported by Mäkinen and Merilä (2008) to the length of our own resulted in the loss of only four terminal haplotypes (see S5). Furthermore, over 25% of these haplotypes were observed in two or more individuals, suggesting the high level of new haplotypes was not due to sequencing error. Thus, of the 117 haplotypes in Britain and Ireland, 107 were unique to the region and not previously described in the literature (GenBank Accession No KC478175-KC478281, see S5 for a complete list). This represents 54% of the total genetic diversity in sticklebacks described across Northern Europe (Mäkinen and Merilä 2008 – 90 haplotypes; Lucek et al. 2010 – 4 haplotypes; this study – 107). High diversity may in part be due to the high number of fish sequenced (*n* = 364) from a comparatively smaller region. While resampling our dataset using a sample size similar to that of Mäkinen and Merilä (2008) reveals a lower mean value of 69 unique haplotypes (SD = 3.97, *n* = 172, based on 10,000 permutations), this still represents 42% of reported diversity. Basic genetic indices further reflect the high diversity of stickleback populations in the region (Table 1). Tajima's D and Fu & Li's F were negative for all mitochondrial lineages (*P* < 0.05), indicating recent population expansion.

**Table 1:** Diversity estimates for regional and lineage groupings of three-spined stickleback mitochondrial sequences

	π ± SD	*H*_ind_	*H*_n_	HD ± SD	*k*	Tajima's D	Fu & Li's F
*Regional*							
Ireland	0.0054 ± 0.00013	259	91	0.965 ± 0.004	4.76	−1.84*	−5.16*
England	0.00272 ± 0.00021	58	18	0.882 ± 0.023	2.77	−0.59	−0.76
Scotland	0.004 ± 0.00038	24	5	0.801 ± 0.037	4.12	1.34	1.64*
Wales	0.00323 ± 0.00114	31	7	0.452 ± 0.11	3.33	−0.57	0.43
Britain & Ireland	0.00458 ± 0.00012	380	117	0.9642 ± 0.0036	4.71	−1.94*	−4.97*
*Lineages*							
European	0.00355 ± 0.00009	332	117	0.9543 ± 0.0059	3.65	−2.2**	−5.41*
Irish	0.00154 ± 0.00017	71	17	0.806 ± 0.041	1.58	−2.1**	−2.49*
Transatlantic	0.00207 ± 0.049	78	28	0.781 ± 0.049	2.13	−2.19**	−3.49*
All	0.00472 ± 0.00011	481	162	0.9684 ± 0.0033	4.86	−2.2**	−4.9*

Nucleotide diversity (π), Individuals sampled (*H*_ind_), haplotype number (*H*_n_), haplotype diversity (HD), mean nucleotide differences (*k*), *P < 0.05; **P < 0.01.

**Figure 4:**
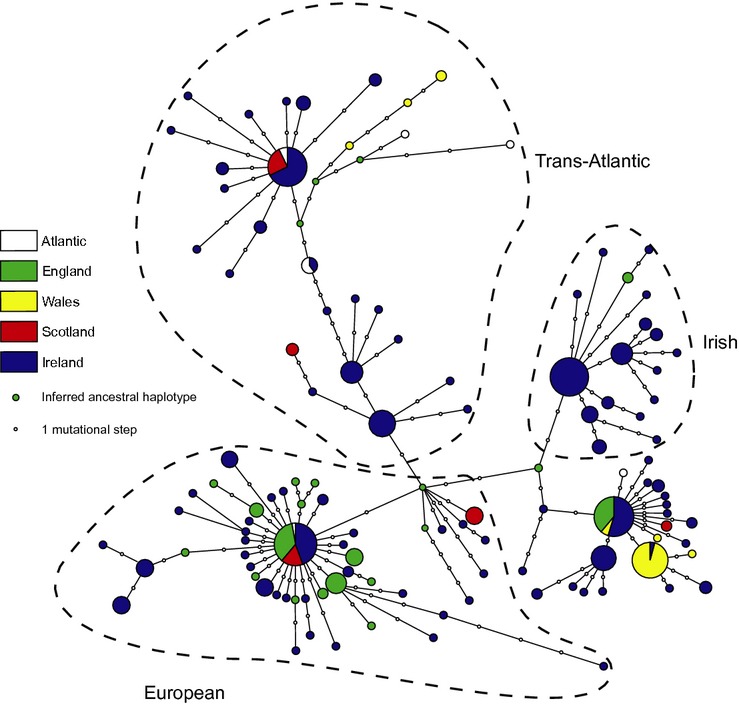
Median-joining network of combined cyt B and control region mitochondrial haplotypes in Britain and Ireland. Lineages are indicated by dashed boundaries.

All *G. aculeatus* sequences formed a monophyletic group relative to *G. wheatlandi*, and a split between Pacific Ocean and Atlantic clades was also highly supported (posterior probabilities = 1.0), consistent with the findings of Ortí et al. (1994). For the British and Irish haplotypes, a strong split was observed between the Trans-Atlantic and European lineages, with the former being basal, most likely representing a more ancient split (Fig. 3). Phylogenetic analysis of previously reported haplotypes alongside the British Isles dataset (Fig. 3) showed a monophyletic lineage with low support within the European lineage (0.51 posterior probability). This lineage was also present along with an additional monophyletic group, both supported by high posterior probabilities (0.7–0.8) when only BI haplotypes were analyzed (see S6). The main mtDNA clades identified in the Bayesian phylogenetic analysis were confirmed by the median-joining haplotype network (Fig. 4). This indicated the presence of three distinct mitochondrial groups within the British Isles. The typical pattern for each of these groups consisted of a geographically widespread ancestral haplotype with spatially confined terminal nodes, radiating in a starlike pattern, thus further supporting the conclusion of recent population expansion (Fig. 4).

**Figure 5:**
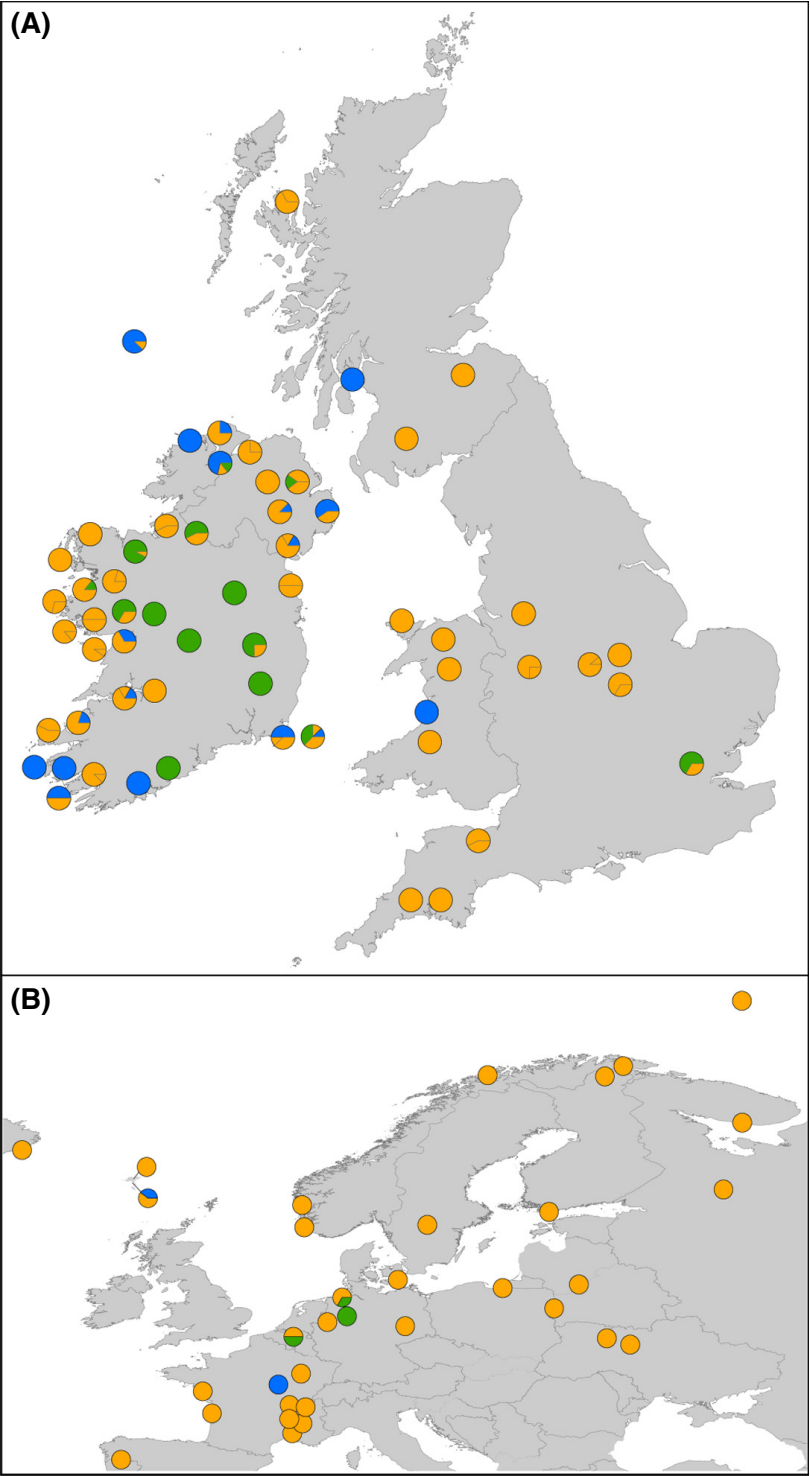
Map of mitochondrial haplotype (cyt B and control region) distribution throughout (A) Britain and Ireland and (B) across Northern Europe and Eurasia. Lineages are represented by color; Transatlantic = blue, European = gold, Irish = green.

The geographical distribution of lineages across Europe and in the British Isles (Fig. 5) suggests that one lineage (herein Irish lineage, see Discussion) is confined almost entirely to South and Central Ireland (Fig. 5A). Furthermore, these haplotypes are rare across Europe, occurring only in low frequencies elsewhere (Fig. 5B). Two additional and more widespread lineages (the European and Trans-Atlantic respectively, see Fig. 5) were also present across Ireland and also in Britain.

### Divergence time estimates and historical demography

**Figure 6:**
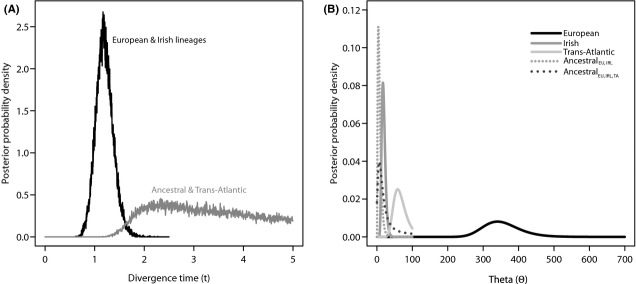
Posterior probability distributions for (A) divergence time and (B) θ parameter estimates generated using combined cyt B and control region sequences for coalescent simulations in IMa2.

Parameter estimates from coalescent genealogy sampling using the isolation-with-migration model (IM) varied in reliability. The majority of estimates produced unimodal posterior probability distributions (Fig. 6) and 95% highest probability density (HPD) estimates within the bounds set by the priors. However, the divergence time estimate for the split between the European/Irish ancestral population and the Trans-Atlantic lineage did not produce a clear posterior density (Fig. 6A) and should therefore be interpreted with caution. Coalescent estimates suggest a divergence time (lower-upper 95% HPD) of 22 kyr BP (16–29 kyr BP) for the Irish and European lineages and 58 kyr BP (32–92 kyr BP) for the deeper split with the Atlantic lineage. Estimates of θ varied considerably between lineages (Fig. 6B and Table 2) and among extant lineages, but were highest for the European and smallest for the Irish (354.8 and 18.9, respectively). The posterior probability distributions for migration between lineages all include zero, suggesting that true isolation was most likely between these populations.

**Table 2:** Mean lineage parameter estimates from posterior probability distributions of coalescent genealogy sampling conducted in IMa2. Parameters are theta (θ), time since population split in coalescent units (*t*) and years (*t*_years_), effective population size (*N*_e_), migration in coalescent units (M) and as individuals per generation, per year (m). Parameters converted using a mutation rate of 5.41 × 10^−5^ (per locus yr^−1^)

Lineage	θ	*t*	*N*_e_	*t*_years_
European	354.80 (257.90–460.90)		1 637,542 (1 190,310–2 127,235)	
Irish	18.96 (9.55–29.65)		87,508 (44,077–136,846)	
Trans-Atlantic	64.04 (37.75–95.05)		295,570 (174,231–438,693)	
Ancestral _EU, IRL_	7.47 (0.55–17.55)	1.20 (0.86–1.56)	34,491 (2 538–81,000)	22,154 (15,947–28,819)
Ancestral _EU, IRL, TA_	22.83 (0.00–74.25)	3.15 (1.71–4.99)	105,369 (0–342,693)	58,209 (31,532–92,068)

Migration	*M*	*m* (per year)
IRL to EU	0.010 (0–0.031)	5.5 × 10^−7^ (0–1.7 × 10^−6^)
EU to IRL	0.082 (0–0.251)	4.4 × 10^−6^ (0–1.4 × 10^−5^)
TA to EU	0.009 (0–0.026)	4.6 × 10^−7^ (0–1.4 × 10^−6^)
EU to TA	0.034 (0–0.102)	1.8 × 10^−7^ (0–5.5 × 10^−6^)
TA to EU	0.081 (0–0.247)	4.4 × 10^−6^ (0–1.3 × 10^−5^)
EU to TA	0.034 (0–0.102)	1.8 × 10^−7^ (0–5.5 × 10^−6^)

### Microsatellite analysis of population clustering

**Figure 7:**
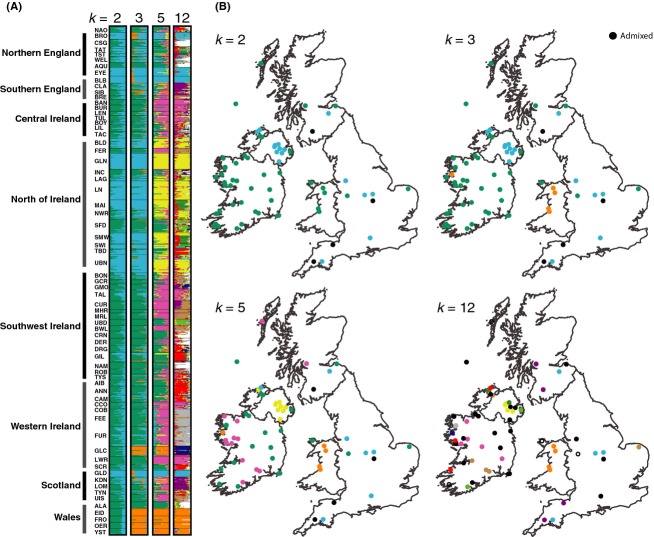
Population clustering estimated using STRUCTURE; (A) individual cluster assignment coefficients grouped by population for best supported clusters of *K* = 2, 3, 5, and 12 based on delta K; (B) spatial distribution of population cluster assignments for *K* = 2, 3, 5 and 12.

Delta *K* values estimated from STRUCTURE analyses provided the highest support for *K* = 19 (see S7), revealing a pattern of clustering among populations located in the same geographical regions (S8). Delta *K* values typically support hierarchical levels of structure (Evanno et al. 2005), and strong support (i.e., high values of delta *K*) was also apparent for multiple *K* values including *K* = 2, 3, 5 and 12 (S9, Fig. 7). At these lower values, a clear separation between populations in the northern and southern regions of Ireland was apparent (Fig. 7). For *K* = 3, populations from Wales represented a third distinct cluster, but there were no clear geographical distinctions between populations from Ireland and the remainder of Britain. For *K* = 5, populations in western Ireland clustered separately from those situated in the central, southern, and northern parts of the region. At the second highest *K* value (*K* = 12), structuring largely occurred among populations in close geographical proximity. However, a large number of samples could not be clearly assigned and were instead considered admixed compared with *K* = 19, the highest value of K which likely also reflected greater population-level structure.

AMOVA indicated that grouping samples by mitochondrial lineage explained a negligible proportion of the total variance (<1%), suggesting low concordance between the mtDNA and microsatellite datasets and divergence since colonization. Similarly, habitat explained a very low but significant percentage of genetic variance (0.37%, *P* < 0.001). Instead, the AMOVA indicated that population groupings based on geographical proximity explained the greatest proportion of variance. For example, when populations were grouped by marine basin and region (see Table S1), these factors explained 2 and 3% of the genetic variance, respectively (*P* < 0.001 in both cases, Table 3). When populations were clustered based on STRUCTURE runs, all values of *K* explained significant proportions of the variance (> 1%), although higher values accounted for the greatest percentages (4.9 and 5.9%, *P* < 0.001 in both instances). Regardless, across all hypotheses, the largest significant partitioning of variance occurred within (83.5–85.0%) and among populations within groups (12.2–15.5%).

**Table 3:** Analysis of molecular variance using microsatellite data for populations of three-spined stickleback in the British Isles; Φ_CT_ = correlation of random haplotypes within a group with random haplotypes from the total dataset; Φ_SC_ = correlation of diversity of random haplotypes from within a population with that of populations within a group; Φ_ST_ = correlation of haplotypes from within a population to haplotypes drawn at random from total sample (Excoffier et al. 1992)

	Among groups				Among populations				Within populations			
			
	df	%	Φ_CT_	*P*	df	%	Φ_SC_	*P*	df	%	Φ_ST_	*P*
Habitat	3	0.37	0.00	0.005	64	14.76	0.15	<0.001	1380	84.90	0.15	<0.001
Marine drainage	4	2.02	0.02	<0.001	63	13.73	0.14	<0.001	1380	84.30	0.16	<0.001
Lineage	2	0.93	0.00	0.001	50	15.52	0.16	<0.001	1077	83.60	0.16	<0.001
Region	15	3.07	0.03	<0.001	52	12.27	0.12	<0.001	1380	84.70	0.15	<0.001
*K* = 2	1	1.84	0.01	<0.001	66	14.10	0.14	<0.001	1380	84.10	0.15	<0.001
*K* = 3	3	3.45	0.03	<0.001	64	12.83	0.13	<0.001	1380	83.72	0.16	<0.001
*K* = 5	5	2.88	0.03	<0.001	62	12.62	0.13	<0.001	1380	84.49	0.16	<0.001
*K* = 12	12	4.90	0.05	<0.001	55	10.67	0.11	<0.001	1380	84.40	0.16	<0.001
*K* = 19	18	5.94	0.06	<0.001	49	10.30	0.1	<0.001	1380	83.77	0.16	<0.001

### Approximate Bayesian computation

**Figure 8:**
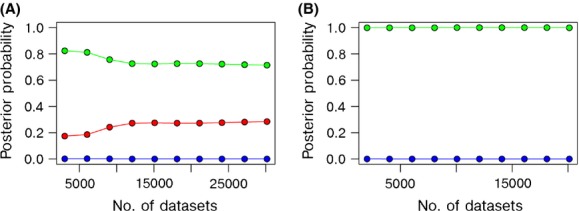
Approximate Bayesian computation posterior probabilities based on combined mitochondrial (cyt B and control region) and microsatellite datasets, calculated using logistic regression for (A) three recolonization scenarios and (B) two scenarios only; red = scenario 1 – colonization from a single source and postglacial divergence of all three lineages; green = scenario 2 – colonization by two sources, European and Transatlantic, Irish lineage diverges from European postglacially; blue = scenario 3 – colonization from three sources, European, Transatlantic, and Irish lineages, all which have diverged prior to the LGM.

ABC analysis strongly supported scenario 2, that is, lineage divergence after the LGM. This was true for analysis using mitochondrial data only (S9a) and combined microsatellite and mtDNA data (Fig. 8). ABC analysis using only microsatellite data supported scenario 3, that is, lineage divergence prior to the LGM (S9b). However, examination of the summary statistics of the simulated datasets revealed that none of the modelled recolonization scenarios fit the observed microsatellite data (S9c). In short, our microsatellite data do not appear to show a signal of recolonization from separate refugia.

In contrast, the logistic regression for the datasets including mitochondrial DNA showed that scenario 2 had the greatest support when all scenarios were compared (mean posterior probability = 0.74, 95% CI 0.47–0.97) and when scenario 1 was excluded (0.99, 0.99–1.00; see Fig. 8). Type I and Type II errors were generally low for all scenarios (<0.30 in all cases, see S10), suggesting that our analyses provided sufficient power to discriminate between models. We interpret our ABC analysis as providing greatest support for scenario 2, that is the Irish lineage diverged from the European in Ireland following the LGM (Fig. 2). Under this scenario, posterior parameter estimates (median, lower and upper 95% confidence intervals) for scenario 2 indicated that the divergence of the Irish lineage occurred 8320 years BP (4950–11,400), while the European and Transatlantic lineages split around the time of the LGM, 14,500 years BP (12,200–25,300). Finally, in concordance with the isolation and migration model, ABC estimated a smaller N_E_ for the Irish lineage (24,800, 15,400–84,800) compared with both the European (1,050,000, 1,010,000–1,660,000) and Transatlantic lineages(100,000, 100,000–103,000, see Table 4).

**Table 4:** Model parameters estimated from prior distributions of scenario 2 (IRL lineage diverged from EU during the Holocene) using approximate Bayesian computation. Parameters are *N* – effective population size for European (EU), Irish (IR), and Transatlantic (TA) lineages, *T* – time since divergence during the Holocene (H) and at the Last Glacial Maximum (L), and μ – mutation rate for mitochondrial DNA and microsatellites, respectively

Parameters	Median	Lower 95%	Upper 95%
*N*_EU_	1.05 × 10^6^	1.01 × 10^6^	1.66 × 10^6^
*N*_IR_	2.48 × 10^4^	1.54 × 10^4^	8.48 × 10^4^
*N*_TA_	1.00 × 10^5^	1.00 × 10^5^	1.03 × 10^5^
*N*_ANC_	4.88 × 10^4^	2.09 × 10^4^	1.16 × 10^5^
*T*_H_	8.32 × 10^3^	4.95 × 10^3^	1.14 × 10^4^
*T*_L_	1.45 × 10^4^	1.22 × 10^4^	2.53 × 10^4^
μ_MTDNA_	6.37 × 10^−8^	3.36 × 10^−8^	8.78 × 10^−8^
μ_MICROSAT_	1.00 × 10^−4^	1.00 × 10^−4^	1.00 × 10^−4^

## Discussion

Regional and geographical variations in glacial history across Europe have influenced patterns of postglacial recolonization and genetic structure in a range of species. Within Western Europe, Ireland has an interesting glacial history, with rapid deglaciation following the LGM, leaving much of the Irish landmass ice-free at ∼19 ka BP; comparatively earlier than other parts of Europe ∼15 ka BP (Ó Cofaigh and Evans 2007; Fretwell et al. 2008). Three-spined sticklebacks occur in a wide variety of aquatic habitats in Ireland (Ravinet et al. 2013) and thus present an excellent test case for determining the effects of the region's glacial history. The results of our study suggest that rapid deglaciation at the southern margin of the British–Irish Ice Sheet shaped the recolonization of Ireland by anadromous fish species, establishing it as a secondary contact zone and likely facilitating the evolution of unique genetic diversity.

### Genetic diversity and structure in a secondary contact zone

High genetic diversity in Ireland and Britain compared with other parts of Northern Europe is consistent with the observation of multiple divergent mitochondrial lineages in stickleback populations. Our analyses confirm previous findings by Mäkinen and Merilä (2008) that haplotypes from the European and Trans-Atlantic lineages, as well as haplotypes from a previously undescribed lineage, co-exist in Ireland and Britain. Secondary contact in this region has been observed in other anadromous fish species, such as brown trout and Atlantic salmon (Consuegra et al. 2002; McKeown et al. 2010). This suggests the location of Ireland and Britain on the western fringe of the Northern Europe, and the pattern of ice retreat following the LGM has facilitated recolonization from multiple sources.

Our microsatellite results did not show the same pattern of divergent lineages. Mitochondrial lineage explained less than 1% of the total genetic variance, and ABC results indicated that hypothesized recolonization scenarios were not appropriate for microsatellite data (S9c). In contrast, microsatellites appear to reflect hierarchical structuring from broad geographical groupings to individual populations. For example, at *K* = 3, STRUCTURE showed a separation between southern regions of Ireland, Britain, and some Welsh populations, while AMOVA indicated that these groups explained a greater proportion of genetic variance (2–4%, see Table 3). Higher *K* values demonstrated more fine-scale geographical clustering and in turn explained a greater proportion of genetic variance (i.e., 6% for *K* = 19). This kind of geographical relatedness has been also observed in other European stickleback studies, although it is typically weak in comparison with the among-population component (Mäkinen et al. 2006; DeFaveri et al. 2012b). This weak geographical clustering likely arises due to a combination of shared ancestry from colonization and contemporary gene flow within geographical regions (Mäkinen et al. 2006).

Although our STRUCTURE results indicate a broad geographical signal, it is likely that the true value of *K* cannot be determined as the number of markers and samples used are insufficient to resolve separate populations effectively (Vaha and Primmer 2006). However, additional support for high values of *K* (i.e., such as *K* = 12 and *K* = 19) and AMOVA indicated that genetic variance was greatest at the population level (10–15%, see Table 3). As previously noted, our sampling strategy was not designed for population-level analyses (Takezaki and Nei 1996; Ruzzante 1998), and thus, between-population variance is likely to be inflated. Nonetheless, these findings are consistent with other stickleback studies using larger numbers of individuals (Mäkinen et al. 2006; DeFaveri et al. 2012b). Drift and isolation of freshwater populations is the most likely explanation for strong divergence at the population level for sticklebacks in Ireland (DeFaveri et al. 2012b).

Discordance between microsatellite and mitochondrial datasets is not uncommon in fish (Lu et al. 2001; Mäkinen et al. 2006; Mäkinen and Merilä 2008; DeFaveri et al. 2012a; Dibattista et al. 2012). For three-spined stickleback, male-mediated gene flow, substitution rate, and effective population size differences may account for this discordance (Cano et al. 2008; DeFaveri et al. 2012a, 2012b). Our results support the suggestion that mitochondrial DNA reflects stickleback evolutionary history throughout the late Pleistocene, whereas more variable microsatellites better reflect postglacial divergence and contemporary gene flow among populations (DeFaveri et al. 2012a).

### Did a cryptic Irish refugium exist for three-spined stickleback?

Our results reveal a previously undescribed mitochondrial lineage in three-spined stickleback, geographically constrained mainly to isolated freshwater populations in Southern and Central Ireland. It should be noted that our reanalysis of the Mäkinen and Merilä (2008) dataset indicates that putatively Irish haplotypes are found at low frequencies elsewhere in Northern Europe (Fig. 5). In each case, however, these are freshwater populations located at relatively close distances to the marine environment and were probably established as a result of long-distance dispersal, which is known to have occurred in sticklebacks (Ortí et al. 1994; Mäkinen and Merilä 2008). Similar long-distance dispersal events also likely explain the presence of Trans-Atlantic haplotypes in Irish coastal populations. Given this, we have designated our additional lineage as being putatively Irish. However, we recognize that further fine-scale work on stickleback mitochondrial phylogeography in Northern Europe is required to confirm that this lineage is limited mainly to Ireland.

Assuming these haplotypes do represent a putatively Irish lineage, our results suggest that at face value, a cryptic Irish refugium could have played role in postglacial recolonization of sticklebacks in the region. A cryptic Irish refugium has previously been hypothesized as the cause of unique diversity in aquatic populations in Ireland (Ferguson et al. 1978; Provan et al. 2005; Maggs et al. 2008). For example, McKeown et al. (2010) suggested that two refugia, in the Celtic Sea and to the west of the Irish landmass, may have played a role in recolonization of brown trout. Under a similar hypothesis for sticklebacks, an Irish lineage would have diverged from the European lineage around the time of the LGM, persisting in an isolated refugium with a comparatively small effective population size. However, the results of our approximate Bayesian computation approach were not consistent with these expectations and instead supported a scenario where the Irish lineage arose due to isolation *following* the last glaciation, probably during the late Pleistocene and early Holocene (i.e., 5–11 kyr divergence time for the Irish lineage). Thus, for three-spined stickleback, it seems unlikely that isolation in an Irish refugium during the LGM can explain unique genetic diversity in the region.

Nonetheless, unique Irish diversity in sticklebacks and other anadromous species suggests that Ireland's glacial history has shaped genetic structure in some way. On the basis of our findings, we suggest that further consideration should be given to the nature of a cryptic refugium in Ireland. A major complication for reconstructing colonization history in Ireland has been uncertainty over the glacial history of the region. Using the traditional model of LGM ice extent in Ireland, several studies have suggested that a refugium may have occurred for both terrestrial and aquatic fauna in the Kerry Peninsula to the south-west (Sinclair et al. 1999; Krebes et al. 2010). Recent data, however, suggest that this area was under ice during the last glaciation and was unlikely to have served as a refugium (Ó Cofaigh and Evans 2007; Scourse et al. 2009; Clark et al. 2012; see Fig. 1). Nonetheless, unique genetic diversity, particularly in anadromous or marine species, may have persisted in a cryptic refugium located in close proximity to the Irish landmass (Provan et al. 2005; Maggs et al. 2008; Provan and Bennett 2008). During the last glacial, sea levels were approximately 120 m lower and thus coastlines would have extended far beyond their present limits (Lambeck 1995; Lambeck and Chappell 2001; see Fig. 1). While some studies have suggested that a refugium may have occurred off Ireland's western coast (Maggs et al. 2008; McKeown et al. 2010), this seems unlikely as recent evidence from chronologically well-constrained shallow marine sediment profiles suggests the BIIS reached the extent of the coastal shelf during the LGM (Scourse et al. 2009; Clark et al. 2012). In contrast, while the southern extent of the BIIS may have reached as far as the Scilly Isles, some of the continental shelf would likely have been exposed at this margin (Toucanne et al. 2009; Clark et al. 2012; see Fig. 1 of this study for margin extents).

It is possible then that exposed coastline to the south of Ireland could have acted as a refugium for some anadromous and marine species. However, exposed coastline at the southern margin of the BIIS was unlikely to have been isolated from the rest of the Atlantic Basin (Fig. 1); therefore, there is still a need to explain why unique diversity occurs in Irish populations. For species with relatively low dispersal potential such as marine gastropods, unique diversity may have arisen due to isolation by distance (Mäkinen et al. 2008; Panova et al. 2011). This is unlikely to have occurred for three-spined sticklebacks which show very little genetic structuring in marine populations (Mäkinen et al. 2006; DeFaveri et al. 2012b). However, rapid deglaciation of the southern margin of the BIIS left much of Ireland ice-free by 19 kyr BP (Ó Cofaigh and Evans 2007; Clark et al. 2012; Ó Cofaigh et al. 2012). For other parts of Britain and Ireland, ice sheet re-advance and low sea levels meant that recolonization could not occur until later in the Pleistocene (Lambeck 1995; Scourse et al. 2009). Recolonization of deglaciated southern areas could have occurred quickly from exposed coastline, and freshwater populations may have even been established at retreating ice-margins (Von Hippel and Weigner 2004). As deglaciation continued, further sea-level change may have isolated these freshwater populations. Indeed, estimated divergence times between the Irish and European lineages (5–11 kyr BP) are consistent with estimates for the geographical isolation of Ireland from Britain and Continental Europe by rising sea levels at the onset of the Holocene (Lambeck 1995). This process of early recolonization and then subsequent postglacial isolation may also explain why glacial relict species such as the Irish pollan occur only in Ireland and not elsewhere in Britain and the rest of Europe (Harrod et al. 2002; Ferguson 2004).

## Conclusion

Regional variation in glacial history has shaped the distribution of genetic diversity across Northern Europe as a result of postglacial recolonization. Patterns of unique genetic diversity in multiple taxa from Ireland suggest the possibility of recolonization from a cryptic refugium. However, in light of recently updated glaciological models, it is essential to test the hypothesis that isolation in a refugium close to Ireland occurred during the LGM. Our study shows that while a unique lineage found largely in Ireland may appear to be evidence of the role of an Irish refugium for three-spined sticklebacks, observed divergence time parameters do not support this hypothesis. Instead, rapid deglaciation at the southern margin of the British–Irish Ice Sheet may have facilitated rapid recolonization to some parts of Ireland well before colonization occurred in the rest of the British Isles. Further analysis is now needed to explicitly test the question of an Irish refugium in other species and to see whether our updated hypothesis of recolonization can explain unique haplotypes in Irish populations of these taxa. With an increasingly well-resolved glacial history, and rapid postglacial isolation, Britain and Ireland represent an excellent testing ground for these questions.
